# A novel pathogenic germline mutation in the adenomatous polyposis coli gene in a Chinese family with familial adenomatous coli

**DOI:** 10.18632/oncotarget.4776

**Published:** 2015-07-30

**Authors:** Shan-Shan Jiang, Jian-Jun Li, Yin Li, Long-Jun He, Qi-Jing Wang, De-Sheng Weng, Ke Pan, Qing Liu, Jing-Jing Zhao, Qiu-Zhong Pan, Xiao-Fei Zhang, Yan Tang, Chang-Long Chen, Hong-Xia Zhang, Guo-Liang Xu, Yi-Xin Zeng, Jian-Chuan Xia

**Affiliations:** ^1^ Sun Yat-Sen University Cancer Center, State Key Laboratory of Oncology in South China, Collaborative Innovation Center for Cancer Medicine, Guangzhou, China; ^2^ Department of Endoscopy, Sun Yat-Sen University Cancer Center, State Key Laboratory of Oncology in South China, Guangzhou, China

**Keywords:** *APC* gene, familial adenomatous polyposis, exon deletion, targeted next-generation sequencing, chinese population

## Abstract

Familial adenomatous polyposis (FAP) is an autosomal dominant disease manifesting as colorectal cancer in middle-aged patients. Mutations of the adenomatous polyposis coli (*APC*) gene contribute to both FAP and sporadic or familial colorectal carcinogenesis. Here we describe the identification of the causative *APC* gene defects associated with FAP in a Chinese pedigree. All patients with FAP were diagnosed by their combination of clinical features, family history, colonoscopy, and pathology examinations. Blood samples were collected and genomic DNA was extracted. Mutation analysis of *APC* was conducted by targeted next-generation sequencing, long-range PCR and Sanger sequencing. A novel mutation in exon 14–15(c.1936_-_2148 del) and intron 14 of the *APC* gene was demonstrated in all FAP patients and was absent in unaffected family members. This novel deletion causing FAP in Chinese kindred expands the germline mutation spectrum of the *APC* gene in the Chinese population.

## INTRODUCTION

Familial adenomatous polyposis (FAP) is an autosomal dominant inherited disease characterized by the presence of numerous adenomatous polyps in the colon and rectum. If not diagnosed and treated early, affected individuals can develop colorectal cancer (CRC) at a mean age of 40 years. The incidence of FAP is approximately 3–10/100,000 [1]. Many extra-colonic manifestations are also seen, including congenital hypertrophy of the retinal pigment epithelium, osteomas, dental abnormalities, and upper gastrointestinal polyps (in the stomach or duodenum) amongst others [2]. Two FAP phenotypes have been described [3]: the classical form (CFAP), defined as >100 colorectal polyps and early onset (polyp formation in the second decade of life); the attenuated form (AFAP) with 10–100 colorectal polyps and a 10–25-year delay onset of adenomatosis and CRC compared with CFAP [4].

The adenomatous polyposis coli (*APC*) gene is a tumor-suppressor gene that is implicated in both FAP and sporadic or familial colorectal carcinogenesis. It is localized to chromosome 5q21-22 and comprises 16 exons. The most common transcript (NCBI RefSeq: NM_001127510) contains 15 coding exons and 1 upstream non-coding exon. More than three-quarters of the gene consists of coding sequence, with an open reading frame translated into a 2843 amino acid polypeptide. It is involved in diverse cellular processes, including cell migration and adhesion, transcriptional activation, and apoptosis [5, 6]. The largest exon, exon 16, is the most common site of both germline and somatic mutations. Approximately 94% of *APC* germline mutations are predicted to produce truncated proteins attributed to nonsense or frameshift mutations (small insertions and deletions), mutations in splice sites, deep intronic deletions, or large genomic rearrangements [7].

Previous studies have illustrated the association between *APC* mutations and the phenotype of FAP [8–11]. To date, more than 1000 different *APC* mutations have been found in FAP patients and are registered in the Human Gene Mutation Database (http://www.hgmd.cf.ac.uk/ac). Over 100 of these have been reported in Chinese patients (ZJU-CGGM:http://www.genomed.org/lovd2/home.php?select_db=APC). The numb er of polyps, age of onset, and occurrence of extra-colonic manifestations can be correlated with specific mutation sites.

The identification of an *APC* mutation can predict for a higher morbidity in affected individuals. For this reason, prophylactic colectomy may be considered for affected patients. In the present study, we screened for *APC* gene mutations by targeted next-generation sequencing and long-range PCR, to identify the germline mutations of all affected members in a Chinese FAP family.

## RESULTS

### Clinical evaluation

**Figure 1:**
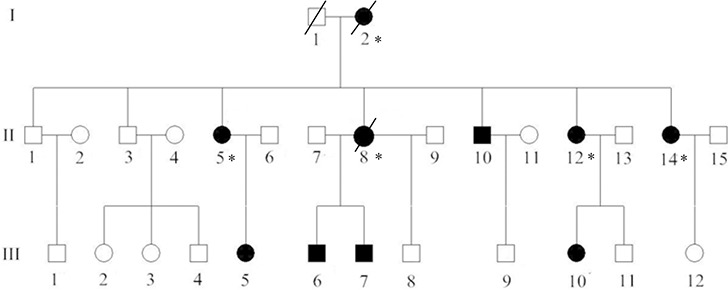
Pedigree structure of the Chinese family with familial adenomatous polyposis Family members with FAP are indicated with Shading. Squares and circles denoted males and females respectively. Individuals labeled with a solidus were deceased. Asterisks refer to colorectal cancer. Roman numerals indicate generations.

We identified one Chinese pedigree with 29 members, of whom ten individuals were affected by FAP. The pedigree suggested an autosomal dominant mode of inheritance (Figure 1). Detailed clinical information for all patients is presented in Table 1.

**Table 1:** Clinical characteristics of 29 index patients found by our study

ID	Family ID	Sex	Exon deletions	Age at diagnosis	Polyp count	CRC (age)	Extra-colonic features
1	I1	M	WT	/	/	/	/
2	I2	F	Del	unspecified	>100	unspecified	None
3	II–1	M	WT	/	/	/	/
4	II–2	F	WT	/	/	/	/
5	II–3	M	WT	/	/	/	/
6	II–4	F	WT	/	/	/	/
7	II–5	F	Del	47	>100	48	None
8	II–6	M	WT	/	/	/	/
9	II–7	M	WT	/	/	/	/
10	II–8	F	Del	36	>100	37	None
11	II–9	M	WT	/	/	/	/
12	II10	M	Del	25	>100	None	None
13	II–11	F	WT	/	/	/	/
14	II–12	F	Del	28	>100	36	None
15	II–13	M	WT	/	/	/	/
16	II–14	F	Del	25	>100	35	None
17	II–15	M	WT	/	/	/	/
18	III–1	M	WT	/	/	/	/
19	III–2	F	WT	/	/	/	/
20	III–3	F	WT	/	/	/	/
21	III–4	M	WT	/	/	/	/
22	III–5	F	Del	25	>100	None	None
23	III–6	M	Del	14	>100	None	None
24	III–7	M	Del	13	>100	None	None
25	III–8	M	WT	/	/	/	/
26	III–9	M	WT	/	/	/	/
27	III–10	F	Del	14	>100	None	None
28	III–11	M	WT	/	/	/	/
29	III–12	F	WT	/	/	/	/

### Strategy for mutation discovery in the *APC* gene

To ensure complete sequencing coverage of all coding regions in *APC*, the quality and reliability of NGS data were evaluated based on the percentage of readable bases and the coverage depth in the targeted region. In the *APC* gene, the coverage depth was up to 200×, with 100% of bases being readable in coding regions. This suggests high capacity for variant identification in most of the exons. Additionally, the mean depth was close to the median depth in each exon, indicating a good randomicity. All novel variations, previously unclassified variations and reported pathogenic mutations detected by NGS were considered as candidate causative mutations and were further confirmed with Sanger sequencing.

### Identification and characterization of candidate mutations

**Figure 2:**
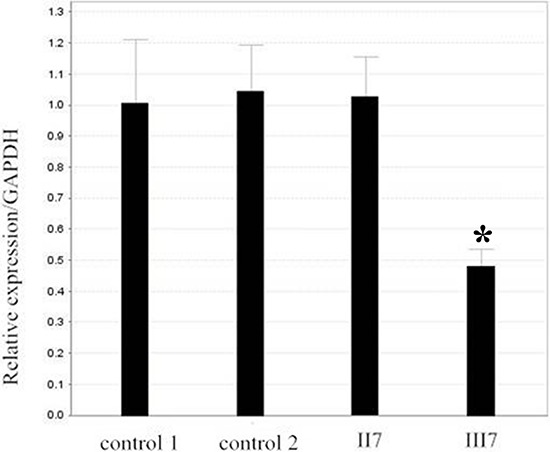
Verification of the deletion in II7 and III7 by real-time PCR Relative amplification (RA) level of the exon 15 in patient II7 and control is nearly 1, and the RA level in III7 is approximately half. GAPDH DNA was used as a loading control.

According to The Human Gene Mutation Database (HGMD), we found that small and large deletions account for approximately 47.2% of abnormalities in patients with FAP. Therefore, in order to identify whether the large deletion was present in III7, who has FAP, we analyzed all exons of the *APC* gene in the son of proband (III7), and his father as a control, by targeted DNA-HiSeq. Through sequencing, we found a deletion mutation in exon 14–15(c.1936_-_2148 del) and intron 14 in patient III7. To further confirm the deletion of exon 14–15 of the *APC* gene in III7, we used quantitative PCR (qPCR) to quantify the DNA copy number of the *APC* gene in the III7, his father (II7) and a normal negative control, with GAPDH being used as a control. Normalizing the amplification of II7 to 1.0, the relative amplification from III7 was approximately 0.47. A normal negative control gave an amplification level of 1.0 (Figure 2). There was a significant difference between III7 and II7 (*P* < 0.05), suggesting that there was a homozygous deletion of exon 14–15 of *APC* in III7. These data suggest that proband (III7) has a deletion of exon 14–15 of the *APC* gene that may be associated with FAP.

### Confirmation of the identified variants by Sanger sequencing in seventeen samples

**Figure 3:**
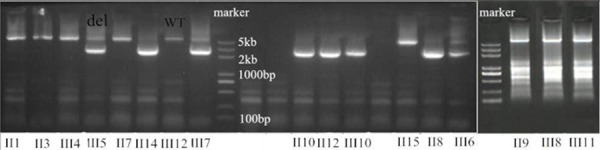
One deletion (exon 14–15 and intron 14) confirmed by long-range PCR An agarose gel shows the PCR products of this deletion in affected members, compared with controls. In the patients, a second fragment is also visible besides the normal product (WT, wild type; del, deletion).

Using the targeted DNA-HiSeq method described above, we identified one gross deletion mutation in one affected patient. To confirm the accuracy of the potential mutations identified by targeted DNA-HiSeq, Sanger sequencing based on long-range PCR was performed. From long-range PCR and sequencing, we found a loss of heterozygosity (LOH) in the *APC* gene of the affected patients. An agarose gel shows a wild type band at 5137 kb, whereas the Del type gives a band of 2.8 kb (Figure 3).

### Genotype–phenotype correlation

The phenotypic spectrum of these *APC* germline mutations is documented in Table 1 and represented schematically in Figure 5.

The deletion mutation has resulted in the loss of either one or both of the â-catenin binding sites, which is crucial for *APC* to decreased â-catenin and prevents the activation of the â-catenin/T cell transcription factor pathway. Patients with these germline mutations exhibit the classical FAP phenotype with more than 100 polyps in the colon. Meanwhile, the Chinese FAP patients with the 212bp deletion at codon 581 have profuse polyps, and none extracolonic manifestations such as osteomas, desmoid and Congenital hypertrophy of the retinal pigment epithelium (CHRPE). Furthermore, the average age of onset of FAP patients with mutation at this codon (25 years) is 14 years earlier than patients with mutation at other sites (39 years) [12]. Three of the eight patients with mutation at codon 581 eventually progressed to colorectal cancer before age 39.

## DISCUSSION

In 1986, Herrera et al. [13] found a new suppressor gene in a 42-year-old white man with Gardner syndrome and colon carcinoma. Groden et al. [14] cloned this gene from colon carcinoma, in 1991, and named it DP2.5 and termed the condition Adenomatous Polyposis Coli. Subsequently, the *APC* gene was implicated in a number of molecular studies of FAP, generating a large volume of data on the mutation spectrum in different countries and ethnic groups [15–17]. Currently, the most up-to-date database of the *APC* gene mutations can be found in the Human Gene Mutation Database, which contains 1207 types of *APC* gene mutation. Small deletions (492 types) comprise the majority, and tend to alter the open reading frame (ORF) and shorten the gene product. The other 334 mutations are missense or nonsense mutations, which create a new STOP codon at the site of mutation. To date, only 190 small insertions have been described. Gross deletions (78 types), mutations in splicing sites (69 types), small indels (25 types), gross insertions (10 types) and complex rearrangements (6 types) were rare. Only three mutations have been described to date in regulatory sequences. Germline mutations are mainly clustered at the 5′ end of the gene, before the mid-point of exon 16. A mutation cluster region (MCR), with a higher frequency of germline mutations, has also been identified in the *APC* gene.

The FAP diagnosis was made following a review of clinical, endoscopic and histological data. The presence of polyposis and the autosomal dominant inheritance mode in this Chinese family, allow the classification of the disease in this family as FAP. It has been reported that FAP accounts for only a small portion of CRCs (about 1%) and is caused mainly by mutations in the *APC* gene [7]. Four CRCs were found with a mean age of 39 years (range 35–37). The actual risk for developing CRC is probably higher than this 37.5%, since a large number of cases may have undergone prophylactic colectomy at a younger age (<30). As in Western studies, Korean FAP patients with the 5 bp deletion at codon 1309 have profuse polyps and extracolonic manifestations, such as osteomas, desmoid, and CHRPE [12]. Furthermore, the average age of onset of FAP manifestations in patients with mutations at this codon is 14 years younger than patients with mutations at other sites (25 years versus 39 years). In our research, the 212 bp deletion at c.1936–2148 in our family manifests with profuse polyps and no extra-colonic manifestations. The mutation with none CHRPE manifestation is at codon 581. This demarcation appears to not be consistent with the findings of an earlier study delineating the CHRPE limits to the region between codons 463 and 1387.

As more germline mutations in the *APC* gene are identified, the molecular mechanisms of FAP become clearer. In this context, searching for an *APC* mutation, especially in patients with a family history of FAP, is important for defining the recurrence risk in their families. Approximately 8–12% of individuals with an *APC*-associated polyposis condition and >100 polyps have a partial or whole *APC* gene deletion [18]. In this study, targeted next-generation sequencing, long range PCR and Sanger sequencing methods were used to complete the mutation analysis of *APC*. The mutation we observed was a 212 bp deletion at c.1936–2148 in patient III7, a gross deletion mutation affecting exons 14–15 of *APC*. According to the frequencies in public databases, this deletion is the putative causal mutation in the family. It was sequenced in the whole kindred to assess the co-segregation of the mutation and the presence of FAP: the mutation was present in eight affected patients, but was absent in unaffected family members. Meanwhile, the variability of the phenotype (e.g. age of onset of clinical manifestations, disease severity) had also been observed in successive generations.

The canonical *APC* protein is a multi-functional molecule, which includes eight known functional sub-domains. The 20 amino acid repeat domain and 15 amino acid repeat domain both engage in β-catenin binding, while SAMP (ser-ala-met-pro) repeats contribute to the axin-binding domain (Figure 5). The ARM domain (armadillo repeat) in the N-terminal region of *APC* binds a variety of proteins, suggesting that *APC* may also be involved in the regulation of cell adhesion, polarization, and migration. ARM-binding partners include the B56 regulatory subunit of protein phosphatase 2A (PP2A) [19]. APC-stimulated guanine nucleotide exchan ge factor (Asef) [20], and kinesin superfamily-associated protein 3 [21]. The deletion at c.1936_-_2148 in exon 14–15 is located in the ARM domain, and creates a STOP codon at residue 581. Mutational inactivation of *APC* leads to the accumulation and nuclear translocation of β-catenin, resulting in aberrant activation of the canonical Wnt signaling pathway implicated in colon cancer development. The ARM domain correlated with multiple proteins including the B56 regulatory subunit of PP2A, reported to both positively and decreased WNT/*β*-catenin signaling [22].

In conclusion, through the investigation of one FAP kindred, a novel gross deletion mutation has been identified in *APC*, which expands the germline mutation spectrum of this gene in the Chinese population. This finding contributes to a more comprehensive database of mutations that could be used for the molecular diagnosis of high-risk mutation carriers, and will in turn inform the use of prophylactic proctocolectomy in FAP.

## MATERIALS AND METHODS

### Ethics statement

The study protocol was reviewed and approved by the Ethics Committee of the Cancer Center of Sun Yat-sen University (Guangdong, China). Written informed consent was obtained from each patient involved in the study.

### Patients and pedigree

**Figure 4:**
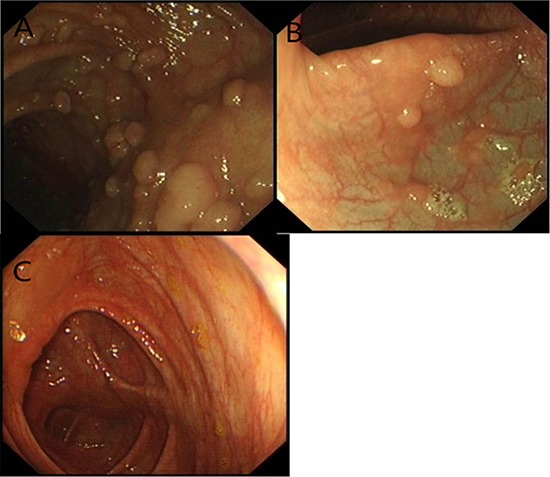
The characterization of the index patients in the pedigree Some sessile polyps were present in the transverse colon. **A.** Tubular adenomas of Patient II8 with focal adenocarcinoma in situ. **B.** Six sessile polyps in the rectum of Patient III6. **C.** No polyps in the unaffected member.

One kindred of FAP patients (Figure 1), diagnosed and treated in the Cancer Center of Sun Yat-sen University, were enrolled in our study. FAP was confirmed in the family by endoscopic screening after patient II8 (proband) presented to Sun Yat-sen University Cancer Center with CRC. The diagnostic criteria were as follows: (1) more than 100 colorectal polyps in total, and (2) at least 20 synchronous adenomatous polyps in patients with a family history of FAP (Figure 4). All patients' clinical information, family history, and the results of colonoscopic, laboratory, and pathologic examinations were collected. We obtained aliquots of 5–10 ml of peripheral blood from as many families members as possible, with full informed consent, and reviewed pathologic slides whenever available.

**Figure 5:**
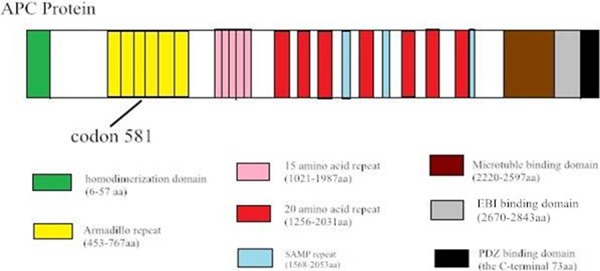
Sequence alignment shows the c.1936–2148 del in exon 14–15 of the APC gene, which creates a STOP codon at 581 Diagram of the protein structure of *APC*.

### Targeted next-generation sequencing and variant identification

A custom capture array (NimbleGen, Roche) was designed to capture all exons, splice sites and the immediately adjacent intron sequences of the *APC* gene that are known to be associated with FAP. The methods used for targeted capture and enrichment, library construction and next-generation sequencing have been previously described [23]. Briefly, genomic DNA was isolated from peripheral blood using a QIAamp DNA Blood MiNi Kit (Qiagen, Hilden, Germany) according to the manufacturer's protocol, and was then sheared to 200–300 bp fragments with an ultrasonicator (Covaris, Massachusetts, USA). The fragments were ligated to oligonucleotide adapters from Illumina (Illumina, San Diego, USA) and successfully ligated segments were amplified by PCR to generate a library using primers that contain the barcode sequence (8 bp) as sample index signature. These were hybridized to a gene-specific array-based chip (NimbleGen, Madison, USA) to capture targeted fragments. After hybridization of sequencing primers, base incorporation was carried out on Illumina HiSeq2000 Analyzers (Illumina, San Diego, USA) to generate paired-end reads (90 bp at each end and 8 bp of the index tag), adhering to the manufacturer's standard cluster generation and sequencing protocols. The image analysis, error estimation and base calling were performed using the Illumina Pipeline (version 1.3.4) to generate raw data. Indexed primers were used to identify the reads from different samples in the raw data.

The 90 bp clean reads were then subjected to alignment with the human reference genome (NCBI database build 37) using the Burrows Wheeler Aligner (BWA) software [24]. Single nucleotide polymorphisms (SNPs) and indels were identified via the SOAPsnp software and GATK Indel Genotyper (The Genome Analysis Toolkit, http://www.broadinstitute.org/gsa/wiki/index.php) respectively. Previously identified SNPs were described in the NCBI dbSNP or HapMap databases. Known disease-causing mutations were identified using the Human Gene Mutation Database at the Institute of Medical Genetics in Cardiff (HGMD, http://www.ghmd.cf.ac.uk/).

### Real-time PCR

To further quantify the DNA copy number change for the *APC* 15 exon in blood from FAP patients, the relative DNA copy number for the *APC* 15 exon was measured by quantitative real-time PCR using an ABI 7900HT Real-time PCR system (Life Technologies, Carlsbad, CA, USA) and HS qPCR Master Mix, according to the manufacturer's instructions. The primers used for amplifying *APC* were: forward, 5′-AAGCGTATTGAGTGCCTTATGG-3′; and reverse 5′- GGTAAGTAAGAGTGCCAACCAA-3′. As a control, the primers used for glyceraldehyde-3-phosphate dehydrogenase (GAPDH) were: forward 5′-CTCCTCCTGTTCGACAGTCAGC-3′; and reverse 5′- CCCAATACGACCAAATCCGTT-3′. The PCR conditions were an initial denaturation step of 95°C for 10 min, followed by 95°C for 10 s, 60°C for 15 s and 72°C for 30 s, for a total of 45 cycles. The relative expression levels of *APC* were normalized to those of GAPDH. The DNA copy number level for the *APC* 15 exon in each sample was compared with the level in control blood samples from normal adult. Data were analyzed using the comparative threshold cycle (2^−ΔΔCT^) method.

### Long-range PCR and Sanger sequencing

To validate the DNA sequence variants detected by Next Generation Sequencing (NGS), we amplified the corresponding gene regions surrounding the variation by long-range PCR, and then sequenced by the resulting products by Sanger sequencing in an ABI 3730 DNA Analyzer (Applied Biosystem, USA). For the variation located at a single copy region (exon 14 to exon 15) of the *APC* gene, primers were designed by Primer 5.0, and synthesized by Invitrogen (Invitrogen Ltd, Shanghai, China) (Table 2).

**Table 2:** Primers for *APC* deletion amplification by PCR

Deletion	Primer location	Primer sequence 5′-3′
Exon 14–15	Exon 14	ACTTA TTTTCCACTGTTCCCCAT
deletion	Exon 16	TTTTGTGCTTTGAATGAATGAGG
